# Quality Assessment of Research Articles in Nuclear Medicine Using STARD and QUADAS-2 Tools

**Published:** 2014

**Authors:** Krisana Roysri, Chanisa Chotipanich, Vallop Laopaiboon, Jiraporn Khiewyoo

**Affiliations:** 1 Department of Biostatistics, Faculty of Public Health, Khon Kaen University, Khon Kaen, Thailand; 2 Department of Radiology, Faculty of Medicine, Khon Kaen University, Khon Kaen, Thailand; 3 National Cyclotron and PET Center, Chulabhorn Hospital, Bangkok, Thailand

**Keywords:** Diagnostic Nuclear Medicine, STARD, QUADAS-2

## Abstract

**Objective(s):**

Diagnostic nuclear medicine is being increasingly employed in clinical practice with the advent of new technologies and radiopharmaceuticals. The report of the prevalence of a certain disease is important for assessing the quality of that article. Therefore, this study was performed to evaluate the quality of published nuclear medicine articles and determine the frequency of reporting the prevalence of studied diseases.

**Methods:**

We used Standards for Reporting of Diagnostic Accuracy (STARD) and Quality Assessment of Diagnostic Accuracy Studies (QUADAS-2) checklists for evaluating the quality of articles published in five nuclear medicine journals with the highest impact factors in 2012. The articles were retrieved from Scopus database and were selected and assessed independently by two nuclear medicine physicians. Decision concerning equivocal data was made by consensus between the reviewers.

**Results:**

The average STARD score was approximately 17 points, and the highest score was 17.19±2.38 obtained by the European Journal of Nuclear Medicine. QUADAS-2 tool showed that all journals had low bias regarding study population. The Journal of Nuclear Medicine had the highest score in terms of index test, reference standard, and time interval. Lack of clarity regarding the index test, reference standard, and time interval was frequently observed in all journals including Clinical Nuclear Medicine, in which 64% of the studies were unclear regarding the index test. Journal of Nuclear Cardiology had the highest number of articles with appropriate reference standard (83.3%), though it had the lowest frequency of reporting disease prevalence (zero reports). All five journals had the same STARD score, while index test, reference standard, and time interval were very unclear according to QUADAS-2 tool. Unfortunately, data were too limited to determine which journal had the lowest risk of bias. In fact, it is the author's responsibility to provide details of research methodology so that the reader can assess the quality of research articles.

**Conclusion:**

Five nuclear medicine journals with the highest impact factor were comparable in terms of STARD score, although they all showed lack of clarity regarding index test, reference standard, and time interval, according to QUADAS-2. The current data were too limited to determine the journal with the lowest bias. Thus, a comprehensive overview of the research methodology of each article is of paramount importance to enable the reader to assess the quality of articles.

## Introduction

Nuclear medicine imaging as well as diagnostic radiological studies including computed tomography (CT) and magnetic resonance imaging (MRI) are important for patient management particularly for making an accurate diagnosis and staging/or restaging of a disease. Even though nuclear medicine studies tend to be less specific, compared to diagnostic radiological imaging, they mostly have high sensitivity, which makes them suitable for early diagnosis, staging, and restaging of diseases.

Some diagnostic nuclear medicine tests are helpful because of their high negative predictive value. Therefore, reporting the prevalence of a disease is essential for helping physicians make decisions based on test results.

The report of the prevalence of a certain disease or condition is very important in studies concerning diagnostic testing, since it affects the positive predictive value (PPV) of a diagnostic test. In fact, a test carried out in a population with a high prevalence of the disease would have a higher PPV, compared to a test performed in a population, where the disease occurrence is rare.

Various radioisotopes are used in nuclear medicine imaging studies so that the patient must receive an appropriate radiation dose. Physicians, who refer patients for nuclear medicine tests, as well as diagnostic radiology, should be concerned about the radiation risks for the patients.

Findings of many studies in diagnostic nuclear medicine provide new insights into this field and help clinicians make decisions for patient management. Standards for Reporting of Diagnostic Accuracy (STARD), as a well-established tool for assessing the value of diagnostic studies, has been adopted by many journals and can be found at www.stard-statement.org. If researchers report their study methods and findings according to STARD checklist, readers will be able to assess the validity of the publication.

Developed from Quality Assessment of Diagnostic Accuracy Studies (QUADAS) (1, 2), QUADAS-2 is used for the assessment of studies, which are planned to be included in the systematic reviews of Cochrane library. This tool focuses on the methodology of a study since the value of study results is dependent on methodology. This checklist assesses the presence of bias (high/low/unclear), although it does not appraise the results or discussion section.

The quality of reporting in diagnostic studies is evaluated by STARD checklist (3-10). Some systematic review articles also use STARD for bias assessment (11), whereas QUADAS is applied to evaluate the reporting of specific diseases in systematic reviews (11-15). Some articles are assessed by both STARD and QUADAS tools (16-18) to determine the quality of research. In fact, both tools can help readers evaluate the quality of a certain article.

Reference standard is of high significance in diagnostic studies. In clinical imaging studies, readers must be familiar with gold standards for each specific disease (such as histopathology report, angiogram, and culture). Some studies may use other imaging modalities, follow up with the same study or other methods.

Research articles published in journals with high impact factors usually have high quality; therefore, readers may be inclined to use the information of a certain article, based on the impact factor of the journal in which that article is published. Reporting the prevalence of a specific disease, which is an essential part of diagnostic radiology and nuclear medicine research, is sometimes not included in some articles.

This study was carried out to determine the frequency of reporting the prevalence of diseases in nuclear medicine articles. We determined the frequency of reporting disease prevalence in nuclear medicine journals with high impact factor, according to STARD stagnant title, each signal question in QUADAS-2 as well as the quality of reference standard.

## Methods

The journals were sorted according to their impact factors in year 2012, provided by the website: www.medical-journals-links.com/radiology-journals-nuclear-medicine-imaging.php. Then, original and clinical research articles were selected from the Journal of Nuclear Medicine, European Journal of Nuclear Medicine and Molecular Imaging, Clinical Nuclear Medicine, Journal of Nuclear Cardiology, and Nuclear Medicine Communications.

Diagnostic clinical studies, published in 2012, were included in the current study. However, studies with similar objectives and populations were excluded. We searched the articles in Scopus database and limited the results of each journal to studies published in 2012.

The search terms were limited to “sensitivity” and “specificity” in order to obtain comprehensive search results; then, articles consisting of diagnostic nuclear medicine tests were determined and included in the study. Two researchers read the abstracts of the articles separately and selected the diagnostic studies for further evaluation. In case of disagreement, the full article was read and a consensus between the two reviewers was reached.

To evaluate the quality of research articles, we assessed each article and recorded the results in a form including STARD, QUADAS-2, disease prevalence report, and a check list concerning the quality of reference standard.

Descriptive statistics were used to analyze and describe the results. Frequency of each STARD and QUADAS-2 item was reported and mean and standard deviation of STARD scores were also calculated. Analysis of the data was carried out using SPSS version 17, and graphs were generated by Microsoft Excel version 2007.

## Results

**Figure 1:**
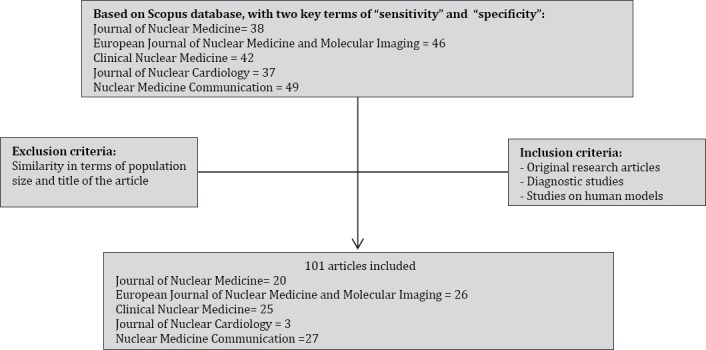
The number of articles in each journal

Our search yielded 212 articles from 5 nuclear medicine journals, among which 101 articles were diagnostic studies. The number of the articles is presented in Figure 1.

Overall, the average STARD score was approximately 17±2.39 points, and the European Journal of Nuclear Medicine had the highest score (17.19±2.38). Some items in the STARD checklist were absent in all journals such as item No.13 (describing the methods for calculating test reproducibility, if done) and No. 24 (reporting the estimates of test reproducibility, if done). Item No. 20 (reporting any adverse events due to performing index tests or reference standard) was found only in the Journal of Nuclear Medicine and Nuclear Medicine Communications. The frequency and proportion of reporting each item in all journals are shown in Table 1, and the average scores are reported in Table 2.

**Table 1:** The presence of each item of STARD checklist (%) in the articles of 5 nuclear medicine journals (CNM=Clinical Nuclear Medicine, EJNMMI= European Journal of Nuclear Medicine and molecular imaging, JNC= Journal of Nuclear Cardiology, JNM=Journal of Nuclear Medicine, NMC= Nuclear Medicine Communications)

	CNM	EJNMMI	JNC	JNM	NMC
1. Identify the article as a study of diagnostic accuracy (recommended MeSH headings: sensitivity and specificity)	23(92.0)	26(100.0)	3(100.0)	20(100.0)	27(100.0)
2. State the research questions or study aims such as estimating diagnostic accuracy or comparing accuracy between tests or across participant groups	25(100.0)	26(100.0)	3(100.0)	20(100.0)	27(100.0)
3. The study population: the inclusion and exclusion criteria and setting and locations where data were collected	25(100.0)	26(100.0)	3(100.0)	20(100.0)	27(100.0)
4. Participant recruitment: Was recruitment based on presenting symptoms, results of previous tests, or the fact that the participants had received the index tests or the reference standard?	25(100.0)	24(92.3)	3(100.0)	20(100.0)	27(100.0)
5. Participant sampling: Was the study population a consecutive series of participants defined by the selection criteria in items 3 and 4? If not, specify how the participants were further selected.	25(100.0)	25(96.2)	3(100.0)	20(100.0)	27(100.0)
6. Data collection: Was data collection planned before (prospective study) or after (retrospective study) performing index test and reference standard?	21(84.0)	25(96.2)	3(100.0)	20(100.0)	26(96.3)
7. The reference standard and its rationale	24(96.0)	26(100.0)	3(100.0)	20(100.0)	27(100.0)
8. Mention technical specifications of materials and methods involved including how and when the measurements were taken and/or cite the references for index tests and reference standard	25(100.0)	26(100.0)	3(100.0)	19(95.0)	27(100.0)
9. Definition of and rationale for the units, cut-offs, and/or categories of the results of index tests and reference standard	23 (92.0)	26 (100.0)	3 (100.0)	17 (85.0)	26 (96.3)
10. The number, training, and expertise of people executing and reading the index tests and reference standard	18 (72.0)	21 (80.8)	3 (100.0)	9 (45.0)	16 (59.3)
11. Determine whether or not the readers of the index tests and reference standard were blinded (masked) to the results of other tests; describe other clinical information available to the readers	12 (48.0)	19 (73.1)	2 (66.7)	9 (45.0)	16(59.3)
12. Methods for calculating or comparing measures of diagnostic accuracy and statistical methods used to quantify uncertainty (e.g., 95% confidence interval)	24 (96.0)	22 (84.0)	3 (100.0)	19 (95.0)	23 (85.2)
13. Methods for calculating test reproducibility, if done	0 (0.00)	0 (0.00)	0 (0.00)	0 (0.00)	0 (0.00)
14. The time of performing the study including the beginning and end of recruitment	15 (60.0)	16 (61.5)	2 (66.7)	13 (65.0)	21 (77.8)
15. Clinical and demographic characteristics of the study population (at least the patients’ age, gender, and spectrum of presenting symptoms)	24 (96.0)	25 (96.2)	3 (100.0)	19 (95.0)	26 (96.3)
16. The number of legible participants, who did or did not undergo the index tests and/or the reference standard; describe why the participants failed to undergo the tests (a flow diagram is strongly recommended)	24 (96.0)	23 (88.5)	2 (66.7)	17 (85.0)	25 (92.6)
17. Time interval between the index tests and reference standard, and any treatment administered in between	9 (36.0)	13 (50.0)	2 (66.7)	9 (45.0)	15 (55.6)
18. Distribution of the severity of the disease (define the criteria) in those with the target condition and other diagnoses in participants without the target condition	19 (76.0)	16 (61.5)	1 (33.3)	10 (50.0)	4 (14.8)
19. Reporting a cross tabulation of the results of the index tests (including indeterminate and missing results) by the results of reference standard; for continuous results, the distribution of the test results by the results of reference standard	19 (76.0)	20 (76.9)	1 (33.3)	19 (95.0)	24 (88.9)
20. Any adverse events due to performing the index tests or reference standard	0 (0.0)	0 (0.0)	0 (0.0)	2 (10.0)	3 (11.1)
21. Estimates of diagnostic accuracy and measures of statistical uncertainty (e.g., 95% confidence interval)	9 (36.0)	11 (42.3)	0 (0.0)	6 (30.0)	8 (29.6)
22. How indeterminate results, missing data, and outliers of the index tests were handled	0 (0.0)	1 (3.8)	0 (0.0)	2 (10.0)	0 (0.0)
23. Estimates of the variability of diagnostic accuracy between subgroups of participants, readers, or centers, if done	4 (16.0)	5 (19.2)	2 (66.7)	7 (35.0)	4 (14.8)
24. Estimates of test reproducibility, if done	0 (0.0)	0 (0.0)	0 (0.0)	0 (0.0)	0 (0.0)
25. Discussing the clinical applicability of the study findings	25 (100.0)	26 (100.0)	3 (100.0)	20 (100.0)	27 (100.0)

**Table 2:** The average STARD scores of 5 nuclear medicine journals with the highest impact factors

	Clinical Nuclear Medicine	European Journal of Nuclear Medicine	Journal of Nuclear Cardiology	Journal of Nuclear Medicine	Nuclear Medicine Communications
STARD score	17.0 ±2.2	17.2±2.4	17.0 ±1.7	16.9±3.2	16.9±2.1
Methodology (Total=11)	9.2 ±1.3	9.2 ±1.2	9.7±0.6	8.7 ± 1.2	9.0± 1.2
Results and discussion (Total=12)	5.9±1.3	6.0±1.5	5.3±2.1	6.3±2.2	5.8±1.3

**Figure 2:**
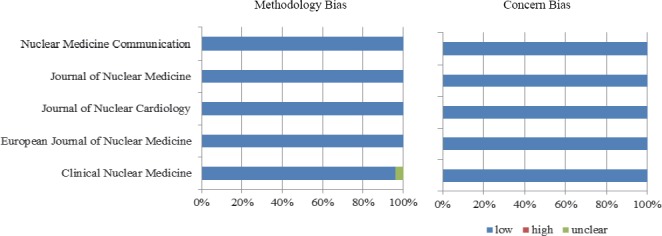
Risk assessment of population, using QUADAS-2

**Figure 3:**
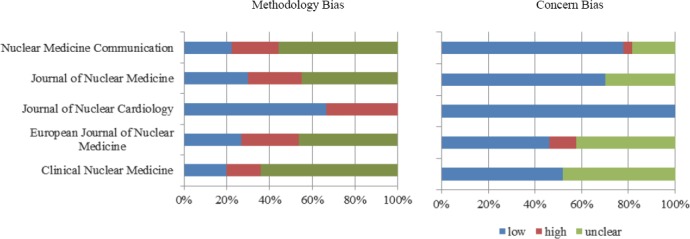
Risk assessment of index test, using QUADAS-2

**Figure 4:**
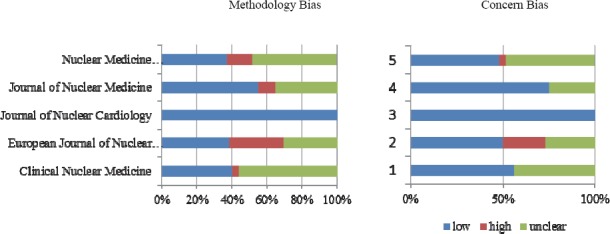
Risk assessment of reference standard, using QUADAS-2

**Figure 5:**
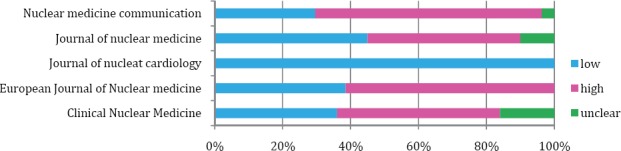
Risk assessment of time interval

According to QUADAS-2 checklist, there was a low risk of bias in many studies of all journals, regarding the study population; however, the index test and reference standard were highly unclear. The European Journal of Nuclear Medicine had the largest number of studies with a high risk of bias regarding reference standard. On the other hand, there was a low risk of bias concerning both reference standard and time interval in articles of the Journal of Nuclear Cardiology. The results obtained from QUADAS-2 tool are shown in Table 3 and Figures 2-5.

**Table 3:** The results of QUADAS-2 for 5 nuclear medicine journals with the highest impact factors

			Clinical Nuclear Medicine (%)	European Journal of Nuclear Medicine (%)	Journal of Nuclear Cardiology (%)	Journal of Nuclear Medicine (%)	Nuclear Medicine Communications (%)
Population	Bias	Low	24 (96.0)	26 (100.0)	3 (100.0)	20 (100.0)	27 (100.0)
		High	0 (0.0)	0 (0.0)	0 (0.0)	0 (0.0)	0 (0.0)
		unclear	1 (4.0)	0 (0.0)	0 (0.0)	0 (0.0)	0 (0.0)
	Concern	Low	25 (100.0)	26 (100.0)	3 (100.0)	20 (100.0)	27 (100.0)
		High	0 (0.0)	0 (0.0)	0 (0.0)	0 (0.0)	0 (0.0)
		unclear	0 (0.0)	0 (0.0)	0 (0.0)	0 (0.0)	0 (0.0)
Index test	Bias	Low	5 (20.0)	7 (26.9)	2 (66.7)	6 (30.0)	6 (22.2)
		High	4 (16.0)	7 (26.9)	1 (33.3)	5 (25.0)	6 (22.2)
		unclear	16 (64.0)	12 (46.1)	0 (0.0)	9 (45.0)	15 (55.6)
	Concern	Low	13 (52.0)	12 (46.2)	3 (100.0)	14 (70.0)	21 (77.8)
		High	0 (0.0)	3 (11.5)	0 (0.0)	0 (0.0)	1 (3.7)
		unclear	12 (48.0)	11 (42.3)	0 (0.0)	6 (30.0)	5 (18.5)
Reference standard	Bias	Low	10 (40.0)	10 (38.5)	3 (100.0)	11 (55.0)	10 (37.0)
		High	1 (4.0)	8 (30.8)	0 (0.0)	2 (10.0)	4 (14.8)
		unclear	14 (56.0)	8 (30.8)	0 (0.0)	7 (35.0)	13 (48.1)
	Concern	Low	14 (56.0)	13 (50.0)	3 (100.0)	15 (75.0)	13 (48.1)
		High	0 (0.0)	6 (23.1)	0 (0.0)	0 (0.0)	1 (3.7)
		unclear	11 (44.0)	7 (26.9)	0 (0.0)	5 (25.0)	13 (48.1)
Time interval		Low	9 (36.0)	10 (38.5)	3 (100.0)	9 (45.0)	8 (29.6)
		High	12 (48.0)	16 (61.5)	0 (0.0)	9 (45.0)	18 (66.7)
		unclear	4 (16.0)	0 (0.0)	0 (0.0)	2 (10.0)	1 (3.7)

The rate of reporting the prevalence of the studied diseases and reference standard are shown in Table 4. The journal with the most frequent reporting of disease prevalence was Nuclear Medicine Communications. The Journal of Nuclear Cardiology had the highest rate of appropriate reference standard (66.7%).

**Table 4:** The report of disease prevalence and the appropriateness of reference standard

		Journal of Nuclear Medicine (N=20)	European Journal of Nuclear Medicine (N=26)	Clinical Nuclear Medicine (N=25)	Journal of Nuclear Cardiology (N =3)	Nuclear Medicine Communications (N=27)
Disease Prevalence Report (%)		21.0	24.0	3.8	0	31.0
Reference standard	Appropriate (%)	36.8	20.0	26.9	66.7	13.8
	Fair (%)	63.2	76.0	69.2	33.3	79.3
	Not appropriate (%)	5.3	8.0	0.0	0	3.4

## Discussion

All journals of clinical nuclear medicine showed similar scores of STARD. This may be related to the researchers’ familiarity with STARD. Some items of STARD were not present or less frequently reported, e.g., reproducibility or adverse effect from the tests. This might be due to the fact that all articles were clinical studies (it is not possible to repeat a certain test on the same subject). Therefore, the details about the adverse effects are not presented since they might be reported to the ethical committee.

Although reporting confidence interval can be helpful for readers in decision-making process, according to statistical findings, the rate of such reports was low (only 42% in European Journal of Nuclear Medicine).

Another important item that should be reported in nearly all articles is describing whether or not the readers of the index test(s) and reference standard are blinded (masked) to the results; however, as the results indicated, the highest rate of reporting was 73%.

According to QUADAS-2 tool, all the studied journal articles were clear in terms of population and sample size. However, regarding index test, a high proportion of studies lacked clarity, e.g., 64% of the articles in Clinical Nuclear Medicine were unclear in this regard; it is not reported in the methodology section. There was also a high risk of bias because the cut-off point for diagnosis was not determined before the results of the standard test were known.

Regarding reference standard, some articles used other imaging modalities, which were not the true reference standard in the study. We found a high risk of bias in 23.1% of the articles in European Journal of Nuclear Medicine; the same was observed concerning time interval. This may be because the authors could not perform an invasive test or had to use more than one single reference standard; the main problem was a negative test.

All journal articles infrequently reported the disease prevalence, e.g., disease prevalence was mentioned only in 31% of the articles in Nuclear Medicine Communication. This may be related to patients’ referral from different parts of the country to nuclear medicine centers; therefore, the authors could not report (or ignored to report) the rate of disease prevalence of a certain disease.

Concerning the reference standard, the articles in the Journal of Nuclear Cardiology used the appropriate reference standard (reported in 66.7% of the articles, since coronary artery catheter is the only reference standard for the diagnosis of coronary heart disease). For other diseases, authors used histopathology, culture, or angiograms to denote a positive finding in the index test. Regarding negative results, researchers might have used other imaging studies or clinical follow-ups as the reference standard.

## Conclusion

The average score of STARD was similar among all five nuclear medicine journals. According to QUADAS-2, there was a low risk of bias in terms of study population. However, there was a lack of clarity in other parts including index test, reference standard, and time interval, due to insufficient reporting the details in the articles.

Overall, STARD can familiarize the readers with the details of methodology and results of a study, and help them decide on the study bias. By using QUADAS-2, readers can know the risk of bias for the methodology as low risk, high risk or unclear from this assessment tool; however, they would not be informed about the bias of the results. We suggest that readers use both tools in the assessment of diagnostic research articles.
